# In Vitro Lactic Acid Bacteria Anti-Hepatitis B Virus (HBV) Effect and Modulation of the Intestinal Microbiota in Fecal Cultures from HBV-Associated Hepatocellular Carcinoma Patients

**DOI:** 10.3390/nu16050600

**Published:** 2024-02-22

**Authors:** Juan Yang, He Gao, Tiantian Zhang, Yong Fan, Yuwei Wu, Xinyu Zhao, Ying Li, Lei Wu, Hui Zhao, Lingshuang Yang, Haojie Zhong, Longyan Li, Xinqiang Xie, Qingping Wu

**Affiliations:** 1 School of Food and Biological Engineering, Shaanxi University of Science and Technology, Xi’an 710021, China; bs1904003@sust.edu.cn (J.Y.); bs210411001@sust.edu.cn (T.Z.); 19822951026@163.com (Y.F.); 2 National Health Commission Science and Technology Innovation Platform for Nutrition and Safety of Microbial Food, Guangdong Provincial Key Laboratory of Microbial Safety and Health, State Key Laboratory of Applied Microbiology Southern China, Institute of Microbiology, Guangdong Academy of Sciences, Guangzhou 510070, China; gaohe@gdim.cn (H.G.); liying@gdim.cn (Y.L.); wuleigdim@163.com (L.W.); zhaohuichinese@163.com (H.Z.); yangls8272@163.com (L.Y.); jaxzhong@126.com (H.Z.); 18868006204@163.com (L.L.); 3 Guangdong Huankai Microbial Co., Ltd., Zhaoqing 526238, China; 13503036950@163.com (Y.W.); zhaoxy9897@163.com (X.Z.)

**Keywords:** lactic acid bacteria, hepatitis B virus, hepatocellular carcinoma, gut microbiota

## Abstract

Hepatocellular carcinoma (HCC), being ranked as the top fifth most prevalent cancer globally, poses a significant health challenge, with a considerable mortality rate. Hepatitis B virus (HBV) infection stands as the primary factor contributing to HCC, presenting substantial challenges in its treatment. This study aimed to identify lactic acid bacteria (LAB) with anti-HBV properties and evaluate their impact on the intestinal flora in HBV-associated HCC. Initially, two LAB strains, *Levilactobacillus brevis* SR52-2 (*L. brevis* SR52-2) and *LeviLactobacillus delbrueckii* subsp. *bulgaicus* Q80 (*L. delbrueckii* Q80), exhibiting anti-HBV effects, were screened in vitro from a pool of 498 LAB strains through cell experiments, with extracellular expression levels of 0.58 ± 0.05 and 0.65 ± 0.03, respectively. These strains exhibited the capability of inhibiting the expression of HBeAg and HBsAg. Subsequent in vitro fermentation, conducted under simulated anaerobic conditions mimicking the colon environment, revealed a decrease in pH levels in both the health control (HC) and HCC groups influenced by LAB, with a more pronounced effect observed in the HC group. Additionally, the density of total short-chain fatty acids (SCFAs) significantly increased (*p* < 0.05) in the HCC group. Analysis of 16S rRNA highlighted differences in the gut microbiota (GM) community structure in cultures treated with *L. brevis* SR52-2 and *L. delbrueckii* Q80. Fecal microflora in normal samples exhibited greater diversity compared to HBV-HCC samples. The HCC group treated with LAB showed a significant increase in the abundance of the phyla Firmicutes, Bacteroidetes and Actinobacteria, while Proteobacteria significantly decreased compared to the untreated HCC group after 48 h. In conclusion, the findings indicate that LAB, specifically *L. brevis* SR52-2 and *L. delbrueckii* Q80, possessing antiviral properties, contribute to an improvement in gastrointestinal health.

## 1. Introduction

Hepatocellular Carcinoma (HCC) stands out as a chronic form with a global mortality rate of 9.1%, ranking it as one of the top fifth most common cancers with respect to human diseases and a serious health burden globally [1]. Hepatitis virus infections, particularly hepatitis B virus (HBV) and hepatitis C virus (HCV), take the lead in impelling the pathological process of viral liver cancer [2]. The gut milieu encompasses various bacteria, eukaryotes, archaea, and viruses, all playing a pivotal role in sustaining the balance and basic functions of healthy hosts through producing active metabolic components [3]. The gut microbiota (GM) can convey different signals to the host’s metabolism, establishing connections between the GM and various organ tissues, hormone levels, and immune systems [4,5]. The gut–liver axis is an interactive pathway established among the intestine and their micro-organisms and the liver by connecting the vena portae, bile duct, and bile secretion system [6,7]. Due to the anatomical specificity of the liver, it is an organ that can easily obtain intestinal toxic factors [7,8]. Moreover, GM dysfunction can elevate intestinal permeability, facilitating the entry of microbial metabolites into the liver, thereby influencing the emergence and evolution of hepatopathy [9,10]. The GM can serve as a biological indicator for clinical features and prognosis of liver diseases [11,12].

Multiple studies have consistently affirmed the secure and effective probiotics in the clinical treatment of various cancers, demonstrating minimal toxicity and rare adverse reactions [13,14]. Probiotics, a type of microbial preparation crucial for maintaining intestinal microbiota stability, predominantly encompass strains of lactic acid bacteria (LAB) such as *Lactobacillus*, *Bifidobacteria*, etc. [15,16]. They actively contribute to accelerating the emergence and course of liver cancer stems because of their ability to regulate the intestinal microbiota [17], stabilize the intestinal barrier (IB) [18,19], and mitigate carcinogenic toxicity [20]. Over the past decades, research on the beneficial health effects of LAB and their active substances has yielded positive outcomes across various domains. 

However, there remains a paucity of research on probiotic therapy specifically tailored for HBV-related liver cancer. Consequently, this study aimed to screen LAB with anti-HBV effects in vitro, simulate the gut environment, regulate the GM of HCC patients, and enhance the overall integrity of the microbiome. This endeavor opens avenues for future adjuvant therapy interventions aimed at decelerating the occurrence of HCC.

## 2. Materials and Methods

### 2.1. Preparation of LAB Fermentation Supernatants (LFSs)

Two *LAB* isolates, including *LeviLactobacillus brveis* (*L. brveis*) SR52-2 (GDMCC 62608) and *LeviLactobacillus delbrueckii* subsp. *bulgaricus* (*L. delbrueckii*) Q80 (GDMCC 62325), were obtained from our previous study and fermented in Li-Mupirocin and Cysteine Hydrochloride Modified de Man, Rogosa and Sharpe (MRS) Medium Base (Guangdong Huankai, Zhaoqing, China) at a 37 °C anaerobic workstation for 24-h. The liquid supernatants were acquired by centrifugation at 4000 rmp for 5 min and with an adjusted pH from 7.2 to 7.4 before storing at −80 °C as LFS stock solution.

### 2.2. Cell Culture

HepG2.2.15 cells (ATCC, Rockville, MD, USA), a human liver cancer cell line, was formed by transfecting HepG cells with a recombinant plasmid containing two connected HBV-DNA genes. It can reproduce asexually in vitro and stably secrete the hepatitis B surface antigen (HBsAg), hepatitis Be antigen (HBeAg), and intact Dane particles in the culture medium for a long time. The cells were cultivated in DMEM (Guangdong Huankai, China) containing 10% (*v*/*v*) FBS (Guangdong Huankai, China), 1% (*v*/*v*) 100 × double antibody (Gibco, New York, NY, USA) and 400 mg/L G418 Geneticin at 37 °C with 5% carbon dioxide (CO_2_) in a humid atmosphere. The culture medium was substituted 2 to 3 times a week.

### 2.3. Cell Viability

The cytotoxicity of LFS on HepG2.2.15 cells was evaluated by CCK-8 reagent (Guangdong Huankai, China) to determine the maximum avirulent concentrations on cells. Briefly, the cells dispersed evenly in the medium were inoculated with a density of 10,000 cells per well in 96-well plates, incubated for 24 h, followed by the addition of 100 μL of fresh DMEM containing different deliquated LFS over 72 h. The CCK-8 reagent was added to each well, according to the instructions. Cell survival rate was determined according to the instructions for the CCK-8. Concentrations of LFS were considered non-poisonous if the corresponding cell viability was in the 100% plus-or-minus-5% range.

### 2.4. Real-Time Quantitative Polymerasechain Reaction (RT-qPCR) Analysis

HepG2.2.15 cells of the same concentration were inoculated onto a 12-well plate. The cells behind the wall were maintained with DMEM containing LFS or lamivudine (LAM, Aladdin, China) for 72 h. Then, cell culture supernatant and cells were used to determine the expression levels of intracellular and extracellular HBV DNA. HBV DNA was extracted using an Aquatic Animal Pathogen DNA/RNA Extraction Kit (Guangdong Huankai, China) and the RT-qPCR was carried out with this primer; the sequence referred to the method of Welzel [21]. RT-qPCR was performed using the Probe qPCR SuperMix (Guangdong Huankai, China). Each experiment group was repeated in triplicate. HBV DNA expression level was calculated using a calibration curve, and the value of untreated cells (positive control) was assigned to 1.00.

### 2.5. Enzyme-Linked Immunosorbent Assay (ELISA)

The expression of extracellular and intracellular HBeAg and HBsAg levels was detected using commercially available ELISA kits (Beijing winter song, China) following the manufacturer’s protocol, by determining the absorbance rate at a wavelength of 450 nm.

### 2.6. Fecal Sample Collection

The work was approved by the Reading’s Research Ethics Committee of the First Affiliated Hospital of Guangdong Pharmaceutical University for Human Study, and was executed following the principles of the Declaration of Helsinki. The samples from healthy individuals must meet the following criteria: (1) normal blood/urine/stool routine examination and hepatorenal functions, (2) no history of liver/kidney disease, and (3) intestinal probiotic and antibiotic prescriptions within 1 month prior to specimen collection. HCC was either diagnosed pathologically or radiologically, according to guidelines for diagnosis and treatment of primary liver cancer in China [22]. All fecal samples of participants were collected with sterile enzyme-free tubes, and immediately transported to a −80 °C refrigerator for storage.

### 2.7. In Vitro Human Fecal Fermentation (HFF)

All samples were thawed at 4 °C; this was carried out as previously described and they were slightly modified in vitro HFF [23]. The difference in the HFF medium consisted in the addition of pure inulin (3 g/L) and 1000 × trace element solution (1 mL/L, MP Biomedicals, Solon, HO, USA). In an anaerobic chamber, the stool samples of HC and HCC groups were well mixed (1/5, *w*/*v*) in normal saline (NS) to prepare the fecal slurry (FS); this was performed to remove the large-particle precipitate by low-speed centrifugation. Prior to the inoculation with the FS (1/10, *v*/*v*), the culture medium was allowed to fit to the ambient conditions of the simulated colon, pH 6.8, and 37 °C. Among them, *L. delbrueckii* Q80 or *L. brveis* SR52-2 strains were added to the fermentation medium, reaching a magnitude of approximately 1 × 10^8^ CFU/mL. Aliquots from each group were taken at the time points 0 h and 48 h, and were promptly refrigerated with liquid nitrogen and stored at −80 °C until assay. Taking equal samples from the HC and HCC group at 0 h and 48 h, they were immediately frozen in liquid nitrogen and stored at −80 °C until assay.

### 2.8. DNA Extraction and 16S rRNA Gene Sequencing

Bacterial genomic DNA was extracted from HFF using the QIAamp DNA Stool Mini Kit (QIAGEN, Hilden, Germany) following the manufacturer’s instructions. 16S rRNA gene sequencing for the V3-V4 region of all extracted DNA was conducted by Shanghai Meiji biological medical technology Co., Ltd. (Shanghai, China). The high-quality sequencing data was subjected to operational taxonomic unit (OTU) clustering analysis. Analyzing the diversity of gut microbiota and the composition of community structure at the phylum and genus levels was carried out, based on taxonomic information.

### 2.9. Gas Chromatography (GC)

The analysis of SCFAs was carried out by referring to the previous research method of our team [24]. In short, peak consistency and quantification were measured using 2-ethyl butyrate as an internal standard, and external standards diluted in 0.3 mol/L 2-ethyl butyrate, dissolved in 0.2 mol/L HCl, were prepared from 400.0 μL of filtered frozen supernatants with stock solutions (Supelco, Bellefonte, PA, USA). The well-mixed liquids were centrifuged at 8000 rmp for 5 min and filtered using 0.22 mm syringe filters. The supernate was transferred into a glass GC vial (Agilent, Santa Clara, CA, USA) and sealed. The standards and samples were measured by GC flame ionization detection (GC-FID) using an Agilent TG-624 SiIMS column (30 m × 0.25 mm ID × 0.25 μm df; Phenomenex). First of all, the baking box temperature was initially kept at 50 °C for 30 s and was subsequently increased at a rate of 10 °C per minute to 140 °C for 30 s. Until the last time it was raised, at a speed of 20 °C/minute to 250 °C for 5 min, the total running time was 20 min. The detector and the injection port were kept at 300 °C and 240 °C, respectively. Each sample being executed contained 1.80 mL of ultrapure water, which was used to wash away any potential carriers. Standards were included in each run to maintain the calibration.

### 2.10. Statistical Analysis

The experimental data was analyzed using Graphpad Prism 7.0 software, and each data set was analyzed using two-tailed Student’s *t*-test (two-sample equal variance). Each experiment was set with three parallels, represented by mean ± standard deviation (m ± SD).

## 3. Results

### 3.1. Characteristics of Subjects

All subject characteristics, including age, gender, and hepatitis B serologic testing, which involves the measurement of several HBV-specific antigens (HBeAg and HBsAg) and antibodies (HBcAb, HBeAb and HBsAb), are presented in Table 1. A total of six participants were included in the study, including three HC and three HCC participants.

### 3.2. In Vitro Screening of Probiotics for Anti-Virus

**Figure 1:**
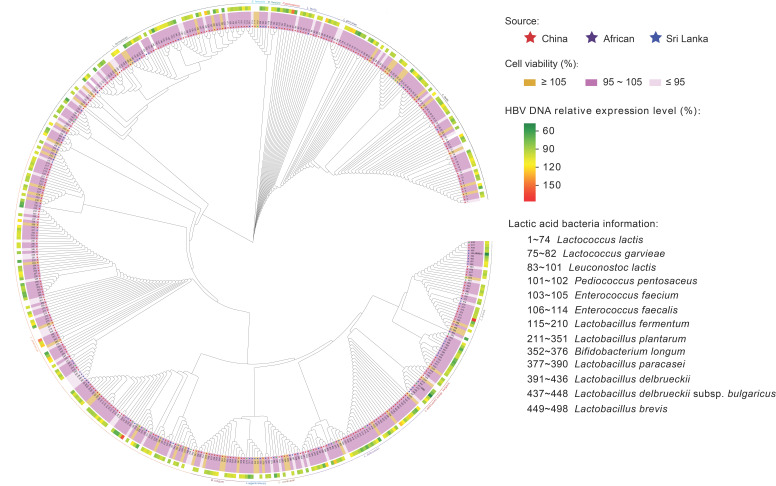
Screening of LAB for antiviral effects of HBV in vitro.

The collection bacterial strains, derived from our team’s prior efforts in bacterial isolation, encompasses 489 strains sourced from different biotopes, including Sri Lanka, Tibet, Inner Mongolia, the Xinjiang Uygur Autonomous Region, and Guangdong province (Figure 1). Initially, employing the CCK-8 assay, we screened the maximum safe concentration of probiotic fermentation broth on HepG2.2.15 cells through a cell activity experiment. This process aimed to exclude cell death resulting from high cytotoxicity concentrations, ensuring that HBV production was unaffected by cell mortality. Cell viability post treatment with LFS revealed that a fermentation broth maintaining 95% to 105% cell activity was chosen for subsequent experiments. Subsequently, cell supernatants were collected following 72 h incubation with the fermentation broth, and HBV DNA was extracted for qPCR quantification. Two LABs, namely *L. brevis* SR52-2 and *L. delbrueckii* Q80, exhibiting potent inhibition of HBV DNA replication, were successfully identified. 

**Figure 2:**
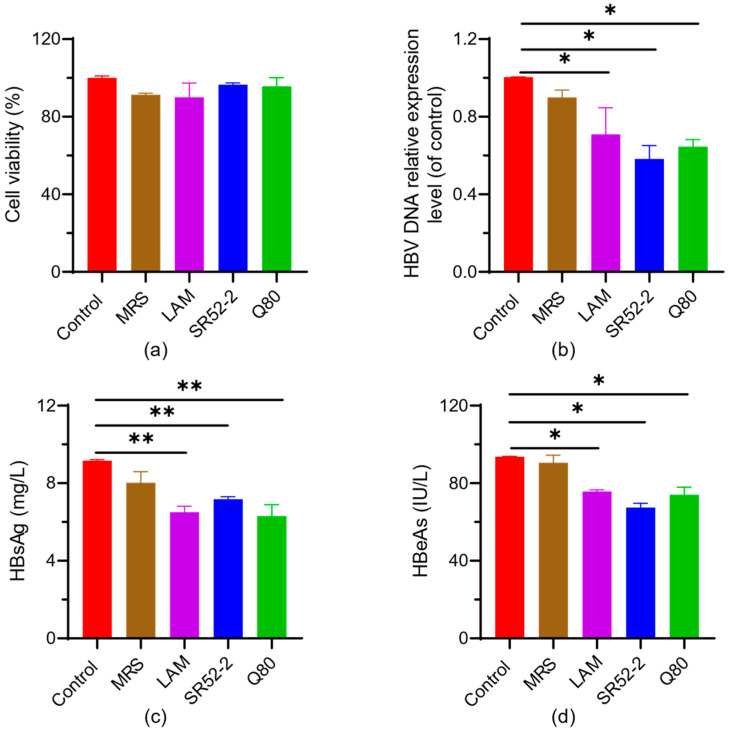
Anti-HBV experiment of LAB. (**a**) Cytotoxicity test; (**b**) HBV DNA extracellular expression level; (**c**) HBsAg extracellular expression level; (**d**) HBeAg extracellular expression level. LAB, lactic acid bacteria; LAM, lamivudine; SR52-2, *L. brevis* SR52-2; Q80, *L. delbrueckii* Q80; * *p* < 0.05; ** *p* < 0.01.

In clinical practice, the infectivity of HBV infection can be assessed based on the level of HBV DNA, HBeAg and/or HBsAg, providing valuable insights into the prognosis of HBV-related diseases. As illustrated in Figure 2b, compared to the control, the relative expression levels of extracellular HBV DNA in *L. brevis* SR52-2 and *L. delbrueckii* Q80 treated groups were 58.23% ± 4.93% (*p* < 0.05) and 64.54% ± 3.06% (*p* < 0.05), respectively. This analysis was conducted within the context of the maximum safe concentration of LFS in HepG2.2.15 cells, ensuring accuracy in HBV DNA expression tests without compromising cell viability (Figure 2a). 

Furthermore, the extracellular expression of HBeAg was observed to be 71.3 ± 2.75 IU/L (*p* < 0.05) and 67.45 ± 1.55 IU/L (*p* < 0.05) for *L. brevis* SR52-2 and *L. delbrueckii* Q80, respectively. Additionally, the extracellular expression of HBsAg was measured at 6.30 ± 0.49 mg/L (*p* < 0.05) for *L. brevis* SR52-2 and 7.18 ± 0.11 mg/L (*p* < 0.05) for *L. delbrueckii* Q80. These findings indicate that both *L. brevis* SR52-2 and *L. delbrueckii* Q80 can inhibit the replication of HBV DNA and the expression of HBeAg and HBsAg, to a certain extent. The application of these strains in antiviral functions merits further investigation. 

### 3.3. SCFAs and pH Profiles during In Vitro HFF

**Figure 3:**
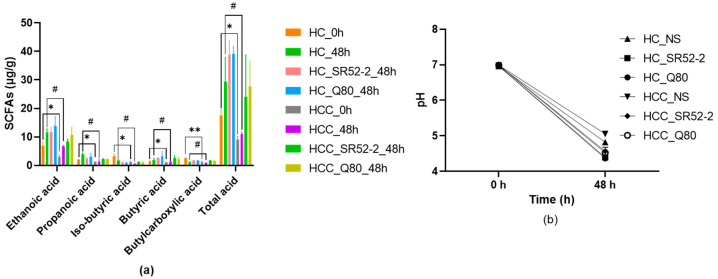
The SCFA (**a**) and pH (**b**) changes in feces during fermentation. HC, health control; HCC, hepatocellular carcinoma; SR52-2, *L. brevi* SR52-2; Q80, *L. delbrueckii* Q80; NS, normal saline; ND, not detected; *n* = 3, mean ± SD; * *p* < 0.05 vs. HC (0-h); ** *p* < 0.01 vs. HC (0-h); ^#^ *p* < 0.05 vs. HC (48-h).

The concentrations of SCFAs in the HFF cultures of HC and HCC at 0 and 48 h, using NS as the control group, were comparatively studied (Figure 3a). The total SCFA concentration increased from 8.97 ± 1.04 mmol/L (0 h) to 10.99 ± 0.64 mmol/L (48-h) in the HCC group (*p* < 0.05). Notably, this value was distinctly higher than that observed in the LAB groups, specifically24.03 ± 11.99 mmol/L and 27.76 ± 7.22 mmol/L in group *L. brevis* SR52-2 and *L. delbrueckii* Q80, respectively, at 48 h (*p* < 0.05).

As depicted in Figure 3b, the trend in pH values mirrored the differences in SCFA concentrations. Consequently, the pH variations were more pronounced in the HC groups than in the HCC groups, with the HCC control group decreasing from 6.99 at the beginning of the process (0 h) to 5.45 after 48 h. Additionally, after 48 h of fermentation with LAB, the pH values of group *L. brevis* SR52-2 and *L. delbrueckii* Q80 in HCC were 4.47 ± 0.10 and 4.52 ± 0.16, respectively.

Further examination focused on the changes in essential SCFA contents in the HCC group. After 48 h of fermentation, the concentrations of acetic acid (8.30 ± 0.74 mmol/L, 10.75 ± 2.06 mmol/L, respectively), propionic acid (2.13 ± 0.20 mmol/L, 2.17 ± 0.19 mmol/L, respectively), isobutyric acid (1.14 ± 0.09 mmol/L, 0.97 ± 0.17 mmol/L, respectively), butyric acid (2.55 ± 0.69 mmol/L, 2.21 ± 0.55 mmol/L, respectively), and valeric acid (0.72 ± 0.02 mmol/L, 0.70 ± 0.02 mmol/L, respectively) in the HCC groups with LAB of *L. brevis* SR52-2 and *L. delbrueckii* Q80 were obviously higher (*p* < 0.05) than those in the 0 h fermentation group (3.08 ± 0.49 mmol/L, 0.88 ± 0.01, respectively). These results indicate that both *L. brevis* SR52-2 and *L. delbrueckii* Q80 can reduce the pH values in the intestines of HC and HCC to varying degrees, increasing the content of short-chain fatty acids.

### 3.4. Effects of LAB on Intestinal Microbiota Diversity

**Figure 4:**
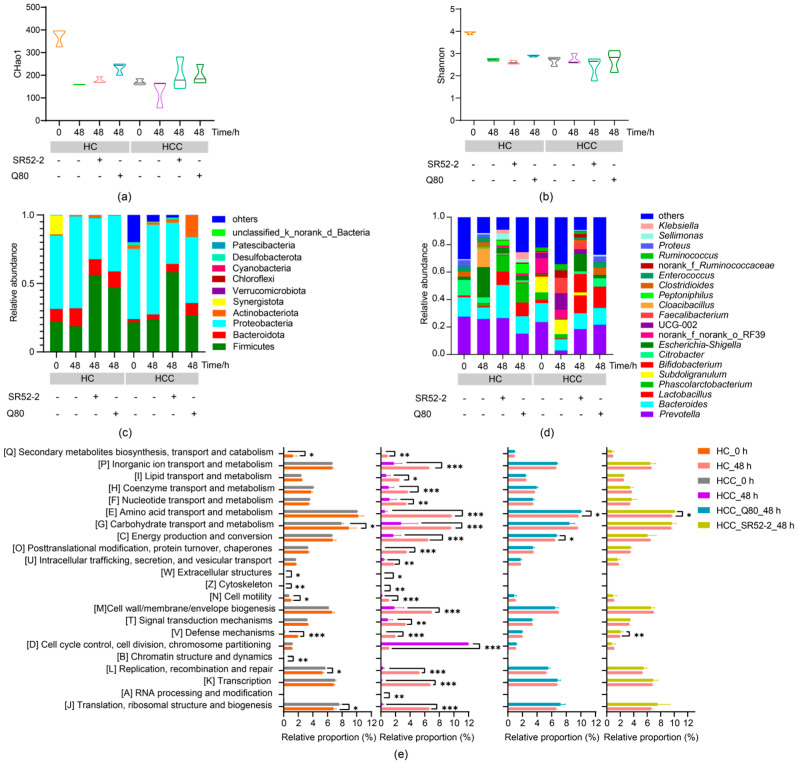
16S rRNA sequencing of GM. (**a**) Chao1 index; (**b**) Shannon index; (**c**,**d**) The community abundance of GM at the phylum and genus level; (**e**) Fecal sample COG classification analysis by using PICRUSt2 functional prediction analysis of GM. HC, health control; HCC, hepatocellular carcinoma; SR52-2, *L. brevi* SR52-2; Q80, *L. delbrueckii* Q80; * *p* < 0.05; ** *p* < 0.01; *** *p* < 0.001.

The intricacy of species diversity within a single sample was assessed using α-diversity indices, including the Chao1 index (Figure 4a) and Shannon index (Figure 4b). In the absence of fermentation, the Chao1 index for the HC group was significantly higher than that of the HCC group. Additionally, community composition analysis was employed to delve into the nuances of microbial variation.

As depicted in Figure 4c, at 0 h of fermentation the HCC group exhibited substantial differences in the phyla Fimucuts, Bacteroidota, Actinobacteriota, and Synergistota compared with the HC group at the phylum level (*p* < 0.05). Following 48 h of fermentation, the alterations in the HCC flora were notably distinct from those of the HC group, especially with the addition of LAB. The abundance of Fimucuts and Bacteroidota at the phylum level significantly increased (Figure 4c, *p* < 0.05). This suggests that the addition of LAB inhibited the growth and reproduction of Proteobacteria, a group primarily composed of Gram-negative bacteria that includes pathogenic strains such as *Escherichia coli* and *Salmonella*.

The relative abundance of general *Lactobacillus*, *Bifidobacterium* and *Bacteroides* in HCC at 0 h was obviously lower than that in the HC group, while for *Escherichia-Shigella* and *Enterococcus* the results were the contrary (Figure 4d, *p* < 0.05). These findings indicated that the abundance of potentially beneficial bacteria in the intestinal flora of the HCC group was lower than that of healthy individuals, and *Escherichia-Shigella* and *Enterococcus* might be implicated in the pathogenic process. 

After 48 h of fermentation, the relative abundance of general *Prevotella*, *Lactobacillus*, and *Bifidobacterium* in the HCC was significantly reduced compared to 0 h, while *Escherichia-Shigella* and *Enterococcus* experienced a significant increase. The relative abundance of *Lactobacillus*, *Bifidobacterium* and *Prevotella* in HCC increased significantly after fermentation with LAB, showing no significant difference from that in the HC group without fermentation. These results suggested that *Lactobacillus*, *Bifidobacterium* and *Bacteroides* may play a pivotal role in the anti-HBV or HCC process, providing valuable insights for subsequent studies on probiotics with anti-HBV and anti-HCC effects. *Escherichia-Shigella* and *Enterococcus* could be considered as potential biomarker targets for the treatment of HBV-induced HCC.

The PICRUSt2 algorithm was employed to predict the functional contributions of the 16S rRNA sequence of genomes. Clusters of Orthologous Groups (COG) based on functional annotation of differentially expressed genes are illustrated in Figure 4e. In comparison to the HC group before fermentation, the GM function in HCC patients displayed significant reductions in seven categories (e.g., secondary metabolites biosynthesis, transport and catabolism, carbohydrate transport and metabolism, cell motility, cytoskeleton, defense mechanisms, extracellular structures, chromatin structure, and dynamics), while two categories (e.g., replication, recombination and repair, post-translational modification, protein turnover, and chaperones) exhibited significant acceleration.

Notably, after simulating the fermentation of the human gut environment for 48 h, the fecal GM function of all HCC patients, except for one classification (cell cycle control, cell division, chromosome division), demonstrated a significant decrease. However, following intervention with *L. brevis* SR52-2 and *L. delbrueckii* Q80 during fermentation, significant differences were observed in three categories (e.g., defense mechanisms, energy production and conversion, and amino acid transport and metabolism) between the HCC group and the HC group. No significant differences were noted in other classifications. This suggests that, during the simulation of the intestinal fermentation process, LAB can contribute to the functional repair of the GM in HCC patients.

## 4. Discussion

The GM profoundly influences human health and contributes to the development of various diseases, including cancer, as extensively demonstrated by numerous studies [25,26]. Moreover, the GM play an important role in determining the efficacy of cancer treatment and the acute as well as long-term toxicity resulting from such interventions [27]. Therefore, maintaining intestinal homeostasis and GM balance is of paramount importance. In the context of HCC patients, the inflammatory pathway primarily drives the progression of liver cancer. This process is instigated by the intricate interplay between intestinal bacteria, the immune system, and the liver, involving interactions among macrophages, Kupffer cells (KuCs), and PAMPs in the liver [28]. The action of PAMPs reaching the liver stimulates immune cells to produce cytokines and chemokines (CCs) through Toll-like receptors (TLRs) and G-protein receptors, thereby promoting inflammation.

HCC, primarily induced by HBV infection, stands as a major global cause of cancer-related mortality [29]. While hepatectomy or transplantation serves as a curative treatment for HCC, it is associated with reduced survival rates and a heightened risk of postoperative recurrence. Considering these limitations, several non-surgical treatment options can be explored for hepatocellular carcinoma. Fecal microbiota transplantation (FMT), dietary interventions, LAB, prebiotics, antibiotics, and bacteriophages have the potential to modulate the GM, thereby enhancing the therapeutic outcomes of HCC.

In the current scientific landscape, numerous studies have underscored the diverse roles of LAB, showcasing their anti-inflammatory [30], antiviral [31], and anti-cancer effects [32]. For instance, our investigation revealed that *Limosilactobacillus fermentum* PV22, exhibited robust antagonism against murine norovirus, reducing viral titers through the production of γ-aminobutyric acid [31]. Fermented milk *Bifidobacterium longum* 070103 can reduce the levels of 3-indole sulfate associated with IB damage [33].

In the realm of anti-HBV treatments, two primary approaches exist: direct antiviral therapy targeting HBV replication and indirect immunomodulatory therapy that enhances cellular immunity to eliminate viral-infected liver cells [1]. Earlier studies reported the inhibitory effects of *Bifidobacterium adolescentis* SPM0212 extract on HBV DNA replication and the reduction of extracellular HBsAg and HBeAg expression levels in HepG2.2.15 cells [34]. However, no probiotics with anti-HBV properties have been reported since then. Currently, leveraging the international resource strain bank platform, our team identified two strains, *L. brevis* SR52-2 and *L. delbrueckii* Q80, which exhibit superior inhibition of HBV DNA replication in vitro compared to the previously mentioned probiotics. In clinical practice, monitoring HBV DNA, HBeAg, and HBsAg levels is critical for evaluating the efficacy of HBV treatment [1,35], as elevated serum levels of these indicators are associated with an increased risk of HCC development and mortality [36].

In our study, the fermentation supernatant of *L. brevis* SR52-2 and *L. delbrueckii* Q80 significantly reduced HBV-DNA levels in HepG2.2.15 cell culture medium by approximately 50%. Although the inhibitory effect was not significantly different from the drug control group, it surpassed that of LAM by about 30%. Complete clearance of serum HBsAg or HBeAg is correlated with minimal recurrence and no progression to HCC in HBV patients. The extracellular levels of HBsAg and HBeAg were reduced by approximately 30% by the fermentation supernatant of both LAB strains in this study, indicating their potent anti-HBV effects at the cellular level.

Under physiological conditions, the GM maintains a delicate equilibrium, forming a dynamic microecological balance crucial for sustaining human health [37]. Imbalance in the GM, characterized by diminished microbial diversity and richness compared to healthy controls, has been well-documented [11,38]. The Chao1 index, reflecting microbial community diversity and richness, and the Shannon index, indicative of community diversity [39], were both significantly lower in HBV-related HCC patients compared to the healthy control (HC) group in this study. These findings highlight a substantial reduction in microbial diversity and richness in HCC compared to HC.

At the bacterial genus level, the relative abundance of potentially beneficial bacteria, including *Lactobacillus*, *Bifidobacterium* and *Bacteroides*, was significantly decreased, while potential pathogenic bacteria such as *Escherichia-Shigella* and *Enterococcus* exhibited a noteworthy increase. Consistent with previous studies, this imbalance in microbial structure and diversity aligns with the observed results. *Escherichia-Shigella* was identified as a potential target microorganism for HCC diagnosis [11]. Traditionally recognized as beneficial, *Bifidobacterium* and *Lactobacillus* contribute to health through various pathways, particularly as major producers of SCFAs [40]. Our team discovered that supplementing these with the potential probiotic *Lactobacillus plantarum* 84-3 increased SCFA content and degraded resistant starch type 3 in vitro, with the combination of acetate enhancing anti-tumor immunity [24]. 

The SCFAs play critical roles in regulating the intestinal environment [41]. Acetic acid, the most abundant GM fermentation product, is absorbed and circulated to peripheral tissues, impacting muscles, heart, and brain [42]. Propionic acid influences gluconeogenesis, immune response, and colonic pH reduction [43,44]. Butyric acid serves as the primary energy source for colon cells, contributing to immune system improvement, oxidative stress reduction, mucin and antimicrobial peptide production, maintenance of epithelial tight-junction integrity, and support of IB function [45]. In the early stages of life, GM finely regulates the liver microenvironment through a butyrate/IL-18-dependent mechanism to ensure the functional maturation of liver-resident natural killer cells [46]. HCC patients, characterized by significantly lower butyrate levels than HC, may benefit from butyrate supplementation to regulate intracellular calcium homeostasis and enhance anti-cancer treatment effectiveness [47]. Valproic acid, produced by *Lactobacillus acidophilus*, binds to the GPR41/43 receptor on liver cell surfaces, inhibiting the oncogenic Rho-GTPase pathway and exhibiting protection against non-alcoholic fatty liver disease-related HCC [48]. In our study, the addition of *L. brevis* SR52-2 and *L. delbrueckii* Q80 for fermentation significantly increased valeric acid concentration in the HCC group, suggesting a potential regulatory effect on valeric acid production.

SCFAs have been reported to delay hepatocellular carcinoma development in HBx transgenic mice [42], while *Enterococcus faecalis*, through its expression of gelE, enhances IB permeability and promotes tumor formation in a TLR4-Myd88 dependent manner [49]. The increase in Gram-negative bacteria, represented by *Escherichia-Shigella* and *Enterococcus*, corresponds to elevated lipopolysaccharides (LPS), triggering local intestinal immune responses and liver inflammation. Considering these insights, targeting the alteration of GM structure, through methods such as supplementing with probiotics, could be a viable strategy for HCC treatment. In this in vitro fermentation simulation, the addition of *L. brevis* SR52-2 and *L. delbrueckii* Q80 increased the relative abundance of potential probiotics in HCC patients’ GM while significantly reducing the abundance of potential pathogenic bacteria. The overall microbial diversity and richness tended towards the HC group. Furthermore, SCFA concentrations in the HCC group were elevated, indicating that additional supplementation with *L. brevis* SR52-2 and *L. delbrueckii* Q80 could contribute to restoring the intestinal microbiota of HCC patients to a more normal state, thereby enhancing antiviral and anti-tumor capabilities. While our findings support the role of potential probiotics with anti-HBV properties in shaping the GM of both normal populations and HCC patients, further research is essential to delineate the effects of L. brevis SR52-2 and *L. delbrueckii* Q80 on the dynamic intestinal environment under diverse conditions and doses.

Multiple functional alterations in the GM of HCC patients were identified through the PICRUSt2 functional prediction analysis method, aligning with the earlier discussed results of the Chao1 index and Shannon index in the HCC group. This correlation suggests a potential link between the reduced species-diversity and richness in the HCC’s GM, particularly those with potential probiotic attributes.

**Figure 5:**
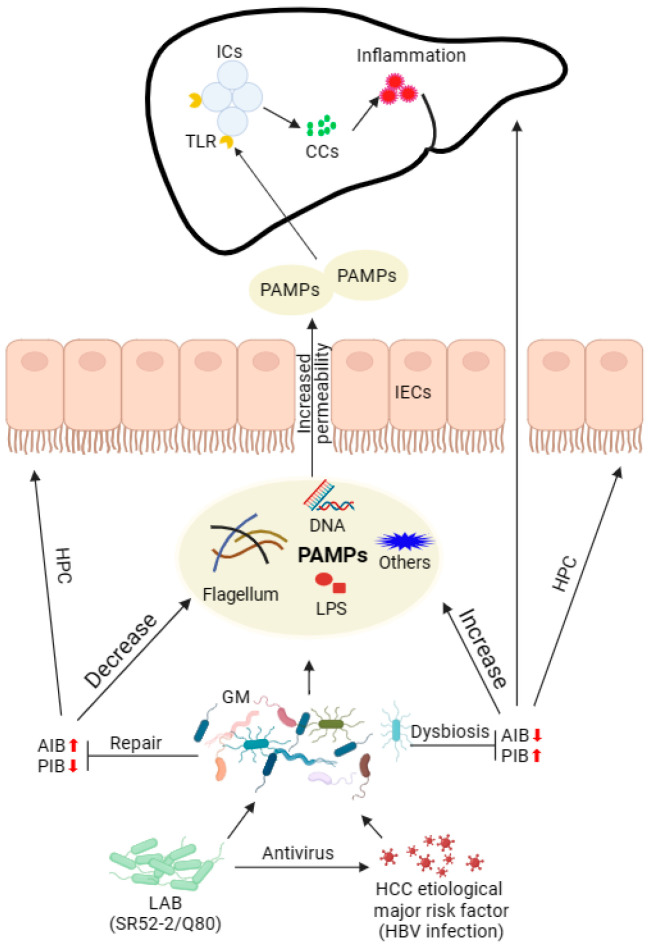
The mechanism of LAB regulating GM, delaying HCC development. Antiviral LAB directly prevents HBV infection and regulates the GM integrity in HCC patients. The integrity of the intestinal epithelium was restored to prevent PAMP leakage into the hepatic portal circulation (HPC), inhibiting PAMPs reaching the liver and limiting TLRs to stimulate immune cells to produce CCs, thereby protecting the liver. Up-pointed red arrows indicate upregulated gene expression/increased pro-inflammatory bacteria (PIB) population; down-pointed red arrows indicate a decrease in the number of anti-inflammatory bacteria (AIB); LAB, lactic acid bacteria; SR62-2, *L. brevis* SR52-2; Q80, *L. delbrueckii* Q80; GM, gut microbiota; IECs, intestinal epithelial cells; PAMPs, pathogen-associated molecular patterns; ICs, immune cells; TLRs, Toll-like receptors; CCs, cytokines and chemokines.

The liver, being the primary organ exposed to GM and its metabolites, encounters circulating metabolites containing PAMPs. These PAMPs are recognized by the bacterial sensor nucleotide-binding oligomeric domain 2 (NOD2), inducing inflammation and nuclear autophagy [50]. This process leads to sustained liver inflammation and hepatocellular damage, and ultimately contributes to the development of HCC. Furthermore, the disrupted ecology of GM facilitates the breakdown of the IB and the subsequent impairment of cellular functions [7]. NOD-like receptors exhibit heightened sensitivity to minimal concentrations of PAMPs, endotoxins, or LPS, triggering an inflammatory cascade that fosters the release of inflammatory factors, accelerating the progression of HCC [51]. It is noteworthy that the intervention of *L. brevis* SR52-2 and *L. delbrueckii* Q80 in the fermentation process in this study resulted in the GM function of the HCC group approaching that of the HC group. This observation indicates a potential role for *L. brevis* SR52-2 and *L. delbrueckii* Q80 in restoring GM function in HCC patients (Figure 5).

## 5. Conclusions

In summary, the two strains of *L. brevis* SR52-2 and *L. delbrueckii* Q80 screened in this study have anti-HBV effects. At the same time, in vitro HFF increased the total content of SCFAs in HCC fecal samples and changed the microbial community structure, resulting in a trend towards the HC group. This indicates that *L. brevis* SR52-2 and *L. delbrueckii* Q80 may exert antiviral effects by producing some active metabolites, and contribute to the diversity and structural remodeling of the GM and functional repair in HCC patients. However, due to the limitations of current research, further research is required to explore the interplay with host health. In conclusion, the two strains, *L. brevis* SR52-2 and *L. delbrueckii* Q80, identified in this study, exhibit anti-HBV effects. Additionally, in vitro HFF led to an increase in the total SCFA content in HCC fecal samples and induced alterations in the microbial community structure, aligning the trend more closely with the HC group. These findings suggest that *L. brevis* SR52-2 and *L. delbrueckii* Q80 may exert antiviral effects through the production of active metabolites. Furthermore, they contribute to the enhancement of diversity, structural remodeling of the GM, and functional restoration in HCC patients. However, given the current research limitations, further investigations are essential to understand the interactions with host health.

## Figures and Tables

**Table 1:** Subject characteristics.

	HC	HCC
Age (years)	49.33 ± 8.18	57.67 ± 11.09
Male/female	2/1	2/1
HBV DNA (IU/mL)	0	352.30 ± 225.85
HBcAb (S/CO)	4.75 ± 3.36	8.98 ± 0.66
HBeAb (S/CO)	1.03 ± 0.72	0.98 ± 0.69
HBsAb (mIU/mL)	155.08 ± 81.55	1.05 ± 1.06
HBeAg (S/CO)	0	9.25 ± 10.63
HBsAg (IU/mL)	0	93.24 ± 111.51

Note: HC, health control; HCC, hepatocellular carcinoma; HBV, hepatitis B virus; HBcAb, hepatitis B core antibody; HBeAb, hepatitis Be antibody; HBsAb, hepatitis B surface antibody; HBeAg, hepatitis Be antigen; HBsAg, hepatitis B surface antigen; *n* = 3, mean ± SD.

## Data Availability

The data presented in this study are available on request from the corresponding author. The data are not publicly available due to the involvement of partners or third parties, which may result in access and usage restrictions.
